# Microorganisms and functional genes in an aerobic-anoxic integrated gold mine wastewater treatment system

**DOI:** 10.1007/s11274-026-04903-3

**Published:** 2026-03-27

**Authors:** Getnet Belay, Carolina Suarez, Addis Simachew, Catherine J. Paul

**Affiliations:** 1 Institute of Biotechnology, Addis Ababa University, P. O. Box 1176, Addis Ababa, Ethiopia; 2 Division of Applied Microbiology, Department of Chemistry, Faculty of Engineering, Lund University, PO Box 124, Lund, SE-221 00 Sweden; 3 Division of Water Resources Engineering, Department of Building and Environmental Technology, Faculty of Engineering, Lund University, PO Box 118, Lund, SE-221 00 Sweden; 4 Institute of Biotechnology, University of Gondar, Maraki 196, Gondar, Ethiopia

**Keywords:** Alkaliphiles, Cyanide degradation, Denitrification, Metagenome-assembled genomes

## Abstract

Biological treatment of cyanide-contaminated wastewater is mediated by microbial consortia in which different organisms perform distinct, functionally specialized roles. This study investigated microbial communities involved in gold mine wastewater treatment with integrated aerobic-anoxic reactors seeded with consortia from an alkaline soda lake, Lake Chitu. Whole-genome sequencing of isolates (WGS) and metagenomic sequencing of the bioreactor were performed to characterize the consortia, resulting in the identification of 23 non-redundant genomes, comprising 14 whole-genome sequencing isolates and 19 metagenome-assembled genomes (MAGs). Most isolated genomes were similar to the recovered metagenomes of MAGs. Except for *Alkalibacterium*, all isolates possessed one or more genes potentially involved in cyanide or cyanate transformation, along with at least one type of terminal oxygenase; however, the gene encoding cynD, which is required for the direct hydrolysis of free cyanide (CN⁻), was not detected. Three representative *Halomonas* isolates harboured the nitrate reductase *narGHI*, nitrite reductase *nirS*, nitric oxide reductase *norB/norC*, and nitrous-oxide reductase *nosZ* genes for full denitrification. All of the isolates possessed several gene clusters associated with different heavy metal resistances. This study suggests that the microbial inoculum sourced from Lake Chitu harbors diverse microorganisms possessing genes potentially involved in cyanide-related metabolic pathways. The findings of this study add to our understanding of the alkaliphilic microbial population that degrades cyanide and cyanide intermediates and provide insight into how these organisms break down cyanide and resist cyanide and heavy metal inhibitory effects.

## Introduction

Gold mining industries release effluents contaminated with cyanide. These effluents usually contain heavy metals like mercury, lead, copper, cadmium, chromium, zinc, and arsenic (Fashola et al. 2016). This polluted effluent is usually stored indefinitely in an artificial dam known as a tailing dam. The tailing dams are built loosely from the surrounding materials and they usually fail due to natural disasters and human-induced factors (Yu et al. 2020). It happened in several countries. Over 18,000 tailing dam failures have been recorded in the last 100 years (Islam and Murakami 2021). The release of tailings before treatment contaminates groundwater and nearby water bodies which causes serious problems for living organisms (Ćwieląg-Drabek et al. 2020; Piciullo et al. 2022). As a result, the gold mining effluent must be treated before being released into the environment. There are various physical, chemical, and biological methods to treat gold mining wastewater. Biological treatment is becoming popular because of its effectiveness, affordability, and ecological friendliness (Alvarado-López et al. 2023).

Some microorganisms can tolerate cyanide toxicity and degrade it into less/no harmful compounds such as ammonia, nitrate, and nitrite (Malmir et al. 2022; Shin et al. 2020). The cyanide concentration, the high pH of the waste, oxygen, and the presence of various co-contaminants can affect the degradation process (Cabello et al. 2018; Khamar et al., 2015). Reactors with appropriate treatment setups provide the opportunity to optimize these variables that have an impact on the treatment process (Denizli and Aslıyüce 2017).

Several bioreactors such as membrane bioreactors (MBRs), fluidized bed reactors (FBRs), discontinuous reactors, packed bed reactors (PBRs), and rotating biological contactors (RBC) (Kumar et al., 2015; Guamán and Nieto, 2018; Shin et al. 2020; Malmir et al. 2022) have been used for biological wastewater treatment. Microbial communities in the reactors can be stabilized and carry out specific, measurable processes, which is important for various purposes (Suarez et al. 2024). Cyanide with other contaminants and microorganisms frequently interact during the treatment process and this interaction results in the production of less harmful substances (Cabello et al. 2018; Newsome and Falagán 2021).

When these reactors are seeded with microbial consortia, several types of microorganisms that perform certain metabolic tasks at different stages of the degradation process are involved (Shin et al. 2020). Various approaches highlight these microorganisms. Some of the frequently identified microorganisms from cyanide-polluted wastewater treatment process include *Pseudomonas*,* Bacillus*, *Alcaligenes*, *Acinetobacter*, *Burkholderia* (Adjei and Ohta 2000; Akcil and Mudder 2003; Kumar et al., 2015; Shin et al. 2020), *Corynebacterium*,* Arthrobacter*,* Thiobacillus*,* Klebsiella*,* Serratia* and *Escherichia* (Ubalua 2010; Kumar et al. 2017; Denizli and Aslıyüce 2017; Guamán and Nieto, 2018; Vallenas-Arévalo et al. 2018) *Agrobacterium* (Potivichayanon 2017), *Providencia*,* Myroides*, and *Proteus* (Mekuto et al. 2018).

Our prior studies on biological treatment of cyanide pollutedwastewater have contributed to our knowledge of cyanotrophic microorganisms (Belay et al. 2025) and the optimal conditions for cyanide-polluted wastewater treatment (Belay et al. 2024). There are also similar studies about the types of cyanotrophic microorganisms and their optimal treatment conditions (Mirizadeh et al. 2014; Safa et al. 2017; Adam and Amankwah, 2020; Jadhav et al. 2022). Microorganisms within the aerobic–anoxic integrated treatment system possess specific metabolic pathways and functional genes that enable them to degrade cyanide when it is the sole nitrogen source. However, a key knowledge gap remains regarding how these microorganisms overcome the inhibitory effects of cyanide on cytochrome *c* oxidases, including the use of alternative respiratory pathways, terminal oxidases with low cyanide sensitivity, detoxification and resistance mechanisms, and metabolic rerouting that enables energy conservation under cyanide stress. The present study aimed to investigate the microbial communities involved in simulated gold mine wastewater treatment, with a particular focus on identifying genes associated with cyanide degradation and transformation, cyanide tolerance and detoxification strategies, heavy metal resistance, and nitrification/denitrification processes.

## Methods

### Operation of the treatment system

A sequential aerobic (2 L)–anoxic (1 L) reactor system was operated at ambient temperature using synthetic gold mine wastewater containing sodium cyanide and acetate as the primary carbon source. The system was inoculated with lake sediment–water slurry and run continuously with an overall hydraulic retention time of ~ 62.5 h. After achieving steady state at 200 mg/L cyanide, operational parameters including cyanide concentration (200–1000 mg/L), flow rate, and carbon source (acetate or glycerol) were varied to evaluate cyanide removal performance. Reactors were maintained under alkaline conditions (pH ≈ 10), with periodic sludge recycling and routine monitoring of cyanide and nitrogen species. The experimental setup, inoculum preparation, and system operation are described in detail elsewhere (Belay et al. 2025).

### Isolation of potential denitrifying and cyanide-degrading strains

The denitrifying bacteria were isolated aerobically on the anoxic denitrificationm (AD) medium (g/L): C_2_H_3_NaO_2,_ 10, KCl, 0.2, FeSO_4_.7H_2_O, 0.1, Na_2_CO_3_, 10, agar 16, and NaCN, 0.2 as the sole nitrogen source at alkaline pH (10.7). At a steady state with approximately 97% cyanide degradation, 10 mL of liquid samples were collected from the reactors. Then, serial dilution in normal saline between 10^− 4^−10^− 6^, 100 µl from the diluted samples were inoculated on the denitrifying media and incubated for 3 to 5 days at 30 °C. After subsequent subculturing, single colonies were picked as potentially denitrifying strains. The isolated strains were confirmed as gas-producing (Li et al. 2008) when grown anaerobically in nutrient broth (Difco) supplemented with 0.15% (NaNO_3_ or NaNO_2_) and 0.1% agar and incubated at 30 °C for 5 days (Desta et al. 2014).

Cyanide-degrading bacteria (CDB) were isolated aerobically on a medium prepared from simulated gold mine wastewater composition. The composition of the medium was (g/L): K_2_HPO_4_.2H_2_O, 3, Na_2_HPO_4_.2H_2_O, 7, MgSO_4_.7H_2_O, 0.3, NaCl, 0.25, CaCl_2_. 2H_2_O, 0.02, Na_2_CO_3,_ 10, CH_3_COONa, 0.25, MnSO_4_. 4H_2_O, 0.3, FeCl_3_. 6H_2_O, 0.045, ZnSO_4_. 7H_2_O, 0.01, CuSO_4_. H_2_O, 0.002, CoCl_2_. 6H_2_, 0.003, NiCl_2_. 6H_2_O, 0.003, NaMoO_4_. 2H_2_O, 0.002 (Alvarado-López et al., 2022; Ingvorsen et al. 1991). The medium also contained 200 mg/L sodium cyanide as the target contaminant, and 16 g/L agar to solidify the medium. The simulated waste components and the agar were mixed with deionized water and autoclaved. Sodium carbonate was autoclaved separately. Similarly, sodium cyanide was prepared by dissolving it in alkaline water and sterilizing it through a 0.2 μm pore size filter paper (polyethersulfone; MilliporeSigma, Burlington, MA, USA).The separately autoclaved sodium carbonate and sodium cyanide were aseptically mixed in the sterilized medium immediately before dispensing into Petri dishes. From a serially diluted sample, between 10^− 4^−10^− 6^, 100 µL sample was spread over the preprepared media and incubated aerobically at 30 °C for 7 days.

The functionality of the isolates in the presence of heavy metals was evaluated by cultivating individual strains in their respective isolation media supplemented with a mixed heavy metal solution (total concentration 145 mg/L: Ni 10 mg/L, Pb 15 mg/L, Zn 40 mg/L, Cu 10 mg/L, Cd 30 mg/L, and Cr 40 mg/L). The selected metal concentrations represent mean levels commonly reported in gold mining–impacted environments.

### Whole genome sequencing

Genomic DNA was extracted from 14 overnight bacterial cultures using the GeneJet Genomic DNA Purification Kit (Thermo Fisher Scientific, MA, USA) according to the manufacturer’s instructions for Gram-negative Bacteria. The concentration of DNA was determined by Qubit dsDNA HS Assay kit using a Qubit 3 Fluorometer (Thermo Fisher Scientific). Library preparation was done with a DNA Prep, (M) Tagmentation (Illumina). Paired sequencing (2 × 300 bp) was done using a NextSeq 2000 System (Illumina) with a 2% PhiX control. Assembly of the reads was done using SKESA v 2.2 (Souvorov et al. 2018). The quality of the genomes was assessed with CheckM2 v.1.02 (Souvorov et al. 2018). CheckM2 v2.3.2 (Chaumeil et al. 2022) was used for the taxonomic classification of the genomes using the GTDB R214 taxonomy (Chaumeil et al. 2022). Bins were dereplicated with dRep v3.4.2 (Olm et al. 2017) using a 95% average nucleotide identity (ANI) threshold. The isolated genomes were annotated with Distilled and Refined Annotation of Metabolism (DRAM) (Shaffer et al. 2020).

### Metagenome sequencing

Shotgun metagenomic sequencing was done for reactor samples to determine if the isolates were representative of the bioreactor communities. Weekly samples were taken for four weeks after bioreactor start-up (days 7, 14, 21, and 42). While this sampling scheme captured broad community trends, the relatively infrequent time points provided limited temporal resolution, which represents a limitation of the present study. About 200 mL of water sample from the bioreactors was taken on each sampling day, and it was filtered via a 0.2 μm filter before being stored at −20 °C. Metagenomic DNA was extracted with the FastDNA spin kit for soil (MP Biomedicals, USA), according to the manufacturer’s instructions, except for the bead beating step which was done twice. The bead-beating step was performed twice because the reactor sludge samples were very thick and viscous, making it difficult to achieve efficient cell lysis with a single round. DNA quantification was done the same as for the isolates. Library preparation for sequencing was with Illumina DNA PCR-free, and paired sequencing (2 × 151 bp) was done in a NovaSeq6000 (Illumina, USA) using a S4 flow cell.

Adapter trimming and quality filtering of metagenome reads were done with fastp v0.23.4 (Chen 2023). Previous assembly reads were normalized to 70x with bbnorm in the BBTools suite v38.61b (http://sourceforge.net/projects/bbmap/). Assembly of reads was done with Megahit v1.2.9 (Shaffer et al. 2020) separately for each sample. For subsequent multi-sample binning, the individual assemblies were combined, followed by read mapping with bwa-mem2 v2.2.1 (Md et al., 2019). Binning of the combined assemblies was done with SemiBin2 v2.1.0 (Pan et al. 2023) using the easy multi-sample binning mode. The quality of the bins was assessed with CheckM2 v.1.02. Bins were dereplicated with dRep v3.4.2 using a 95% ANI threshold and using criteria of 75% completeness and 10% contamination to select MAGs. Taxonomy for the MAGs was determined with GTDB-Tk v2.3.2 and GTDB R214.

### Comparison of isolates and MAGs

FastANI was used to compare isolate genomes and MAGs using ANI (Jain et al. 2018). Isolate genomes and MAGs were assessed to be similar if their ANI was at least 99%.

### Heterotrophic nitrification

After gene prediction with Prodigal, Blast+ (Camacho et al. 2009) was used to search for in both isolate genomes and MAGs for homologs to the dnfABC genes from *Alcaligenes faecalis* for Direct Ammonia Oxidation (Dirammox) to nitrogen gas (Jq et al. 2022).

## Results

### The growth conditions of the pure isolates

Most isolates could grow at alkaline pH on a solid medium supplemented with a mixture of heavy metals. All isolates, except, P-DN R2, isolated as a potential denitrifier were able to utilize NaNO_3_ or NaNO_2_ to grow in anaerobic conditions. The isolate P-DN R2 could not able to grow when NaNO_2_ was the sole nitrogen source (Table 1).

**Table 1:** The alkalinity tolerance, heavy metal resistance, and denitrification activity of the representative isolates

Isolates	Isolation category	Grow in the presence of heavy metals	Denitrification activity when a separate NaNO_3_ or NaNO_2_ was provided as a sole nitrogen source	
			NaNO_3_	NaNO_2_
P-CN-A12	CN^−^degrader	+	*	*
P-CN-AN16	CN^−^degrader	+	*	*
P-CN-AN6	CN^−^degrader	-	*	*
P-DN-B2	Denitrifier	-	+	+
P-DN-D2	Denitrifier	+	+	+
P-DN-I2	Denitrifier	-	+	+
P-DN-P2	Denitrifier	+	+	+
P-DN-R2	Denitrifier	+	+	-

*Not tested

### Isolates studied with a Shotgun WGS

A total of 14 isolates, comprising eight potential denitrifiers, and six cyanide degraders were sequenced. Among the cyanide degraders, two isolates were classified as members of genus *Halomonas*, two as *Actinotalea*, one as *Aliidiomarina*, and one as *Salisediminibacterium*. For the denitrifier group, seven isolates were members of genus *Halomonas*, and one was classified as a member of *Alkalibacterium.*

As indicated in Table 2, after dereplication, eight genomes were selected based on their average nucleotide identity (ANI). The two *Actinotalea* genomes were similar to each other (> 99% ANI). Five of the *Halomonas* isolates were closely related to *Halomonas* sp003182195, and present in both the cyanide degrader and denitrifier group had more than 99% ANI. Two other isolates that were similar to each other (> 98% ANI), and also present in both groups, were named *Halomonas stevensii *(Table 2).

**Table 2:** Classification and genome quality of the representative isolates

Isolate	Closest relatives	Comp	Cont	Mbp	Contigs	Group	Gm type	GC (%)
P-CN-A12	*Aliidiomarina*	99.98	0.05	3.0	62	CN (1)	-	51.6
P-CN-AN16	*Actinotalea*	100	1.36	3.6	257	CN (2)	-	72.6
P-CN-AN6	*Salisediminibacterium*	99.98	0.03	3.2	63	CN (1)	+	48.9
P-DN-B2	*Halomonas* *sp 024137965*	100	0.3	3.4	119	DN(1)	-	67.7
P-DN-D2	*Halomonas*	99.99	0.12	4.3	58	DN (1)	-	64.5
P-DN-I2	*Halomonas sp003182195*	99.98	0.23	3.6	102	DN(4), CN (1)	-	68
P-DN-P2	*Alkalibacterium*	99.37	0.43	2.5	62	DN (1)	+	41.7
P-DN-R2	*Halomonas stevensii*	100	0.39	3.6	183	DN(1), CN (1)	-	60.1

Abbreviations: completeness (Comp), contamination (Cont), GM (Gram type), CN (Cyanide degrader), DN (Denitrifier)

In samples from day 7 and day 14, these 8 genomes matched 49% and 57% of the reactor metagenome reads. For samples from days 21 and 42, they corresponded to 19% and 29% of the reads. These results indicate that isolation successfully recovered a substantial fraction of the microbial community, although the eight genomes do not represent the majority of the community at all time points.

#### Cyanide degradation and denitrification genes

Cyanide dihydratase (*cynD*), which converts cyanide into ammonia and formate (Jandhyala et al. 2003), the fungal cyanide hydratase (*cht*) that produces formamide (Wang and VanEtten 1992) could not be detected in the isolates. Homologs to thiosulfate sulfurtransferase (*rhdA*), that using thiosulfate can produce thiocyanate (Cipollone et al. 2007), were observed in the *Actinotalea*, *Salisediminibacterium*, and *Halomonas* genomes (Sorokin et al. 2001). The *rhdA* likely mediates cyanide transformation/detoxification to thiocyanate rather than full degradation. The potential mechanisms for cyanide degradation in the *Allidiomarina* isolate are unclear, although it is possible that one or more of the genes annotated as the *Pfam* family Carbon-nitrogen hydrolase (PF00795) could be nitrilases. Cyanate lyase (*cynS*) producing ammonia from cyanate was detected for all the *Halomonas* isolates except P-DN-R2.

**Fig. 1:**
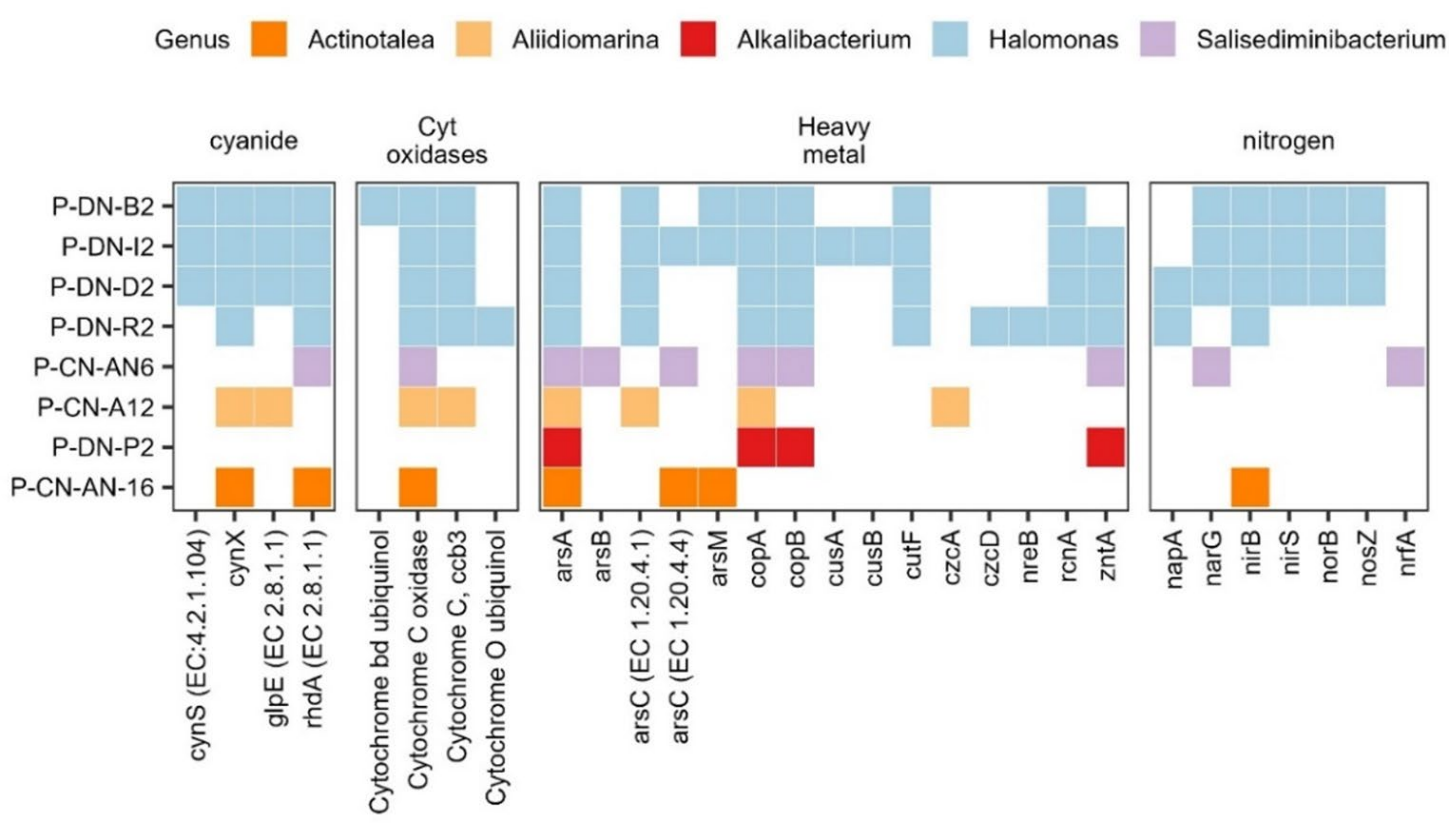
Genes for cyanide degradation, nitrogen cycling, and respiration in representative isolates

Three representative *Halomonas* isolates, P-DN-B2, P-DN-D2, and P-DN-I2, harboured genes for full denitrification. These were the nitrate reductases *narGHI*, nitrite reductase *nirS*, nitric oxide reductase *norBC*, and nitrous-oxide reductase *nosZ*. The *Halomonas* isolate P-DN-R2 lacked most genes linked to denitrification, except periplasmic nitrate reductase *napAB*. The *Aliibidiomarina*, *Actinotalea*, and *Alkalibacterium* isolates lacked all the above-mentioned genes for denitrification. For the Salisediminibacterium isolate only harboured *narGHI* (Fig. 1). Nitrogenases (*nif*, *vnf* and *anf* genes) could not be detected in the isolates´ genomes.

Cytochrome C oxidases are sensitive to cyanide, and therefore we searched if ubiquinol oxidases were present or if terminal oxygenises were absent. Except for P-DN-P2 (*Alkalibacterium*), all seven representative isolates harboured at least one type of terminal oxygenase. Similar to other *Alkalibacterium* spp (Ishikawa et al. 2009), the present *Alkalibacterium* (P-DN-P2) is likely a fermentative bacteria based on the absence of terminal oxygenases, the presence of pyruvate formate lyase, as well as l- and d-lactate dehydrogenases.

All the other seven isolates harboured a cytochrome C oxidase. The high-affinity cytochrome *C ccb3*-type oxidase was present in the *Halomonas* and *Aliidiomarina* isolates and absent in the *Actinotalea* and *Salisediminibacterium* isolates. In addition, P-DN-R2 (*Halomonas stevensii*) had genes for a cytochrome o ubiquinol oxidase and P-DN-B2 (*Halomonas sp*.) harboured all genes for high-affinity bd ubiquinol oxidase. However, all other isolates, except for *Alkalibacterium*, had at least some of the bd ubiquinol oxidase genes (*cyd*AB). The gene *cydX*, however, was absent in all isolates, even though it is essential at least in *Escherichia coli* (Vanorsdel et al. 2013).

#### Heavy metal resistance genes

Genes associated with heavy metal resistance were present in the isolates (Fig. 1). The *arsA* of arsenic and arsenate resistance genes were identified in all our isolates. Similarly, *arsB* was detected in *Salisediminibacterium*. Except for, *Alkalibacterium* and *Salisediminibacterium*, *arsC* was also detected in all isolates. The *copA/B* genes were detected in all isolates except *Actinotalea.* Most isolates except *Actinotalea*,* Aliidiomarina*, and *Halomonas sp024137965* possessed the *zntA* gene.

### Nitrification

Because ammonia might be one of the products of cyanide degradation, nitrification has been proposed as a potential mechanism explaining subsequent nitrite or nitrate production. If followed by denitrification, this would result in nitrogen removal. Neither canonical nitrifiers were observed, nor ammonia monooxygenases could be detected in the metagenome (see below). An alternative to autotrophic nitrification could be heterotrophic nitrification. We searched in the MAGs for genes with homology to those linked to nitrification in *Alcaligenes faecalis*. However, gene clusters with homology to the *dnfABC* gene cluster which are associated with Direct Ammonia Oxidation (Dirammox) to nitrogen gas (Jq et al. 2022) were not observed.

### Metagenome

To identify other microorganisms in the reactor that were not isolated, assembly and binning of the metagenome reads were done. This resulted in 19 MAGs (Table 3), corresponding to around 75% of the reads for days 7, 14, and 42 samples, and 46% from day 21. With seven MAGs, Bacillota was the phylum with the most MAGs, followed by Pseudomonadota with five MAGs.

**Table 3:** Metagenome-Assembled genomes (MAGs)

MAG	Phylum	Closest relatives	Comp.	Cont.	Contigs	Mbp
CYLUAB19	Actinomycetota	g__Bogoriella	100	0.25	39	3.5
CYLUAB1	Actinomycetota	g__Isoptericola	94.73	0.19	353	3.8
CYLUAB13	Actinomycetota	s__*Microbacterium chocolatum*	95.59	4.87	306	3.2
CYLUAB3	Bacillota	s__Bacillus_A paranthracis	100	0	46	5.3
CYLUAB15	Bacillota	s__*Halolactibacillus alkaliphilus*	100	0.37	115	3
CYLUAB12	Bacillota	g__CSBR16-87	81.71	0.09	200	1.4
CYLUAB6	Bacillota	f__Carnobacteriaceae	96.88	0.12	21	2.3
CYLUAB16	Bacillota	f__Carnobacteriaceae	97.55	0.31	64	2.7
CYLUAB11*	Bacillota	g__Alkalibacterium	87.42	0.23	50	2.3
CYLUAB9	Bacillota	s__*Alkalibacterium olivapovliticus*	96.62	2.29	62	2.8
CYLUAB7	Bacillota_A	g__JAFIFG01	100	5.32	196	4.9
CYLUAB10	Bacteroidota	g__Psychroflexus	93.94	0.48	377	2.6
CYLUAB18	Bacteroidota	g__Cyclonatronum	100	0.2	117	3.8
CYLUAB5	Patescibacteria	g__GN02-872	94.86	0.32	20	1.1
CYLUAB2*	Pseudomonadota	g__Aliidiomarina	97.01	0.09	50	3
CYLUAB4*	Pseudomonadota	g__Halomonas	93.49	2.79	35	4.3
CYLUAB8	Pseudomonadota	s__*Halomonas hamiltonii*	82.32	5.24	450	3.1
CYLUAB17	Pseudomonadota	s__*Halomonas nigrificans*	78.64	0.37	38	3.7
CYLUAB14*	Pseudomonadota	s__Halomonas sp003182195	86.56	0.64	87	3.3

A star indicates a MAG that is similar to an isolated genome

Several isolated genomes were similar to MAGs recovered for the metagenome (> ANI 99%). This was the case for the isolates P-DN-D2 and the MAG CYLUAB4 both classified as *Halomonas*. P-DN-I2 was similar to CYLUAB14 (*Halomonas* sp003182195), P-CN-A12 and CYLUAB2 were also similar to *Aliidiomarina*, as well as P-DN-P2 and CYLUAB11 (*Alkalibacterium*). Surprisingly, some of the taxa observed among isolates, such as *Actinotalea* and *Salisediminibacterium*, were not recovered as MAGs.

**Fig. 2:**
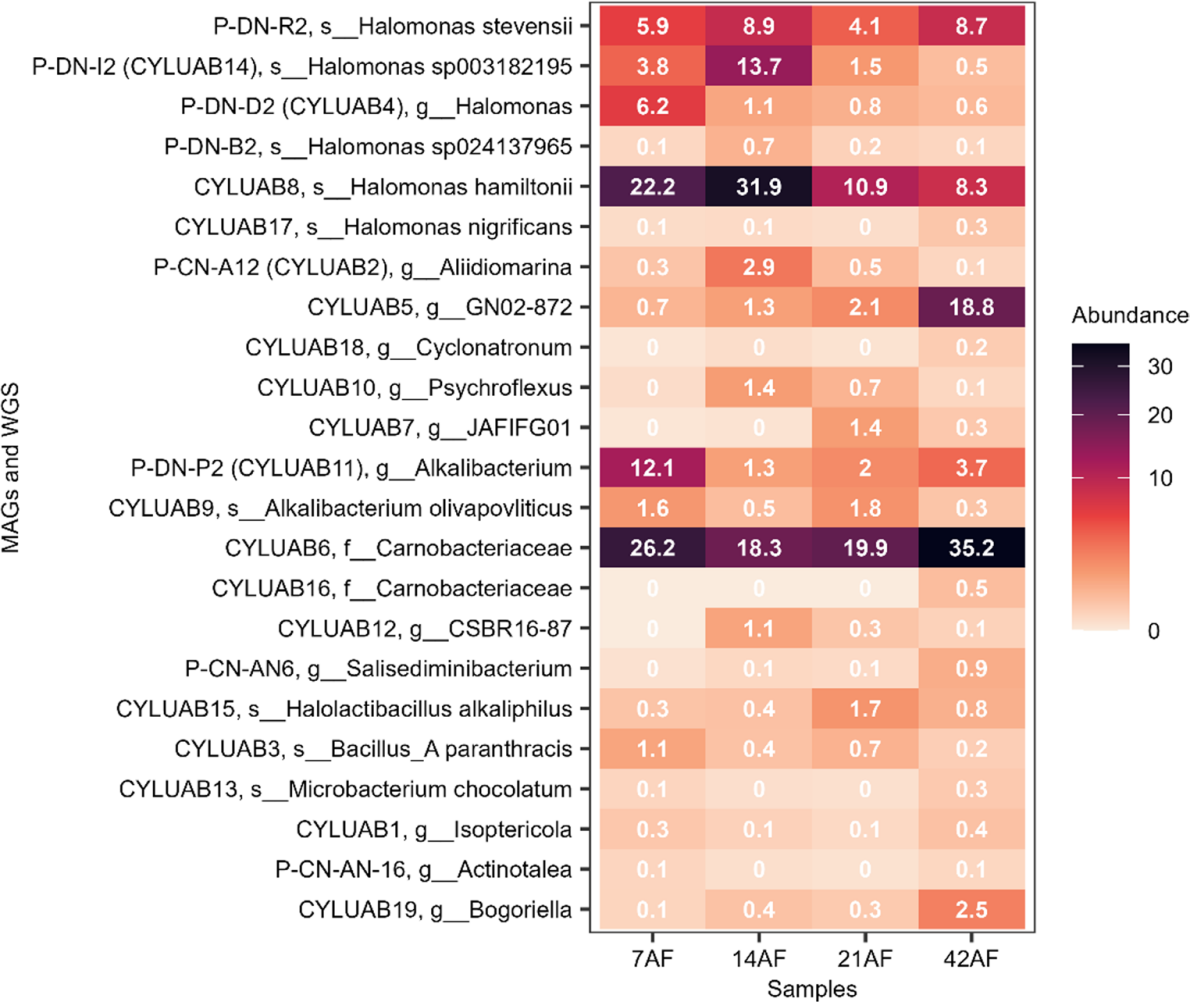
Relative abundance of isolates and MAGs in the reactor metagenome

When combining isolate genomes with MAGs, results in 23 non-redundant genomes (Table 2). For samples from days 7 and 14, this corresponded to 81% and 85% of the metagenome reads, and for days 21 and 42 it was 49% and 83% (Fig. 2).

## Discussion

In the present study, several alkaliphilic microbial communities were identified from the integrated aerobic-anoxic reactors established to treat goldmine wastewater seeded with natural consortia from alkaline soda lakes. Their natural adaptation to the lake’s alkaline environment may help them survive in the reactors under high pH (Belay et al. 2024, 2025). More importantly, the potential to produce various compatible enzymes (Jiang et al. 2012) enables them to degrade cyanide and use the intermediates as a nitrogen source.

The current study used both metagenomics and genome sequencing of isolates to explore the bioreactor community. It revealed 19 MAGs from a total of four metagenome-sequenced samples. Eight non-redundant genomes were found from a total of 14 whole genome sequenced isolates, and the genomes of five of the isolates were comparable (> ANI 99%) to MAGs retrieved from the metagenome. *Bacillota* and *Pseudomonadota* dominated the 23 non-redundant genomes. These two taxa are the most often identified phyla in several cyanide-polluted wastewater treatment plants (Shin et al. 2020) and in the source of our inoculum, the Ethiopian Rift Valley soda lakes(Belay et al. 2025; Jeilu et al. 2022).

This high similarity among MAGs and isolates indicates the accuracy of the assembled MAGs. Similarly, the metagenome reads were aligned with the isolated genomes (49% to 57% for days 7 and 14) and (19% and 29%) for days 21 and 42. This suggests that the isolation technique successfully recovered a significant portion of microbes involved in the treatment process. However, a decrease in genome matching on days 21 and 42, reflects changes in the microbial community composition over time, with some members of the original inoculum decreasing in abundance or being replaced by other taxa during the later stages of treatment. The metagenome assembly and binning successfully revealed microorganisms that were uncovered by traditional culturing. Metagenomics offers advantage in accessing microorganisms that may be difficult to isolate with traditional culturing (Jeilu et al. 2024). However, *Actinotalea* and *Salisediminibacterium*, observed among our isolates, were not recovered as MAGs. The absence of these isolates among the MAGs could be linked to the high GC content, especially for *Actinotalea* (72.9%) affecting the assembly process, or the abundance of these isolates may be below the detection limit for metagenomics analysis.

Most of our isolates possessed several genes linked to cyanide degradation. This suggested the presence of diverse enzymatic pathways that were actively involved in the treatment process. This study suggests that members of the genera *Actinotalea*,* Aliidiomarina*, and *Salisediminibacterium* possess genomic potential for involvement in cyanide-contaminated wastewater treatment, based on the presence of genes putatively associated with cyanide transformation. While these findings expand the current knowledge of microbial diversity harboring cyanide-related functional genes, direct evidence of gene expression or in situ cyanide degradation activity by these organisms was not assessed. The identified genes that are important to encode enzymes include rhodaneses (*RhdA*), nitrilases (nitrile hydratases), and cyanase. However, cyanidase (*cynD*) is one of the bacterial cyanide-degrading enzymes (Luque-almagro et al. 2005; Olaya-Abril et al. 2024) previously detected in various CDBs such as *Pseudomonas pseudoalcaligenes CECT5344* (Roldán et al. 2021) *Alcaligenes xylosoxodans* (Olaya-Abril et al. 2024), and *Bacillus pumilus C1* (Mekuto et al. 2016) could not be detected in any of the present isolates.

Cyanate lyases encoded by the *cynS* gene (Cipollone et al. 2008; Luque-almagro et al., 2018) were detected in three of the four *Halomonas* isolates. The genus *Halomonas* has been shown in earlier studies to be an effective cyanide degrader (Khamar et al. 2015; Sorokin et al. 2001). This enzyme is also reported in other CDBs such as *Rhodococcus* spp (Alvillo-Rivera et al. 2021). Although further investigation is needed to confirm the specific function, in the *Allidiomarina* isolate, one or more genes annotated in the *Pfam* family carbon-nitrogen hydrolase (PF00795) could be nitrilases. Nitrilase hydrolyzes carbon-nitrogen triple bonds (nitrile or cyano groups) producing ammonia and the corresponding carboxylic acid or amide product (Cabello et al. 2018; Park et al. 2017; Zhu et al. 2018). Homologs to thiosulfate/cyanide sulfurtransferase (*rhdA)* were detected in the *Actinotalea*, *Salisediminibacterium*, and *Halomonas* genomes. Prior studies on CDB from *Bacillus subtilis*, *Pseudomonas aeruginosa*, *Azotobacter vinelandii*, and various *Thiobacillus* strains indicated that this enzyme is vital in cyanide degradation pathways (Alvillo-Rivera et al. 2021; Cipollone et al. 2007; Gupta et al. 2010). Furthermore, heterologous expression of *Pseudomonas aeruginosa RhdA* has been shown to increase cyanide tolerance in *Escherichia coli* (Cipollone et al. 2008).

Among the whole genome-sequenced isolates, only *Alkalibacterium* lacked genes linked to known pathways for cyanide degradation. This isolate, however, was able to grow on solid media where cyanide served as the sole nitrogen source. This suggests that *Alkalibacterium* possesses a yet unidentified cyanide degradation mechanism. Understanding this mechanism could pave the way for new approaches to cyanide bioremediation. However, as it lacks terminal oxygenase but has pyruvate formate lyase and l- and d-lactate dehydrogenases, which are common in other fermentative *Alkalibacteria* such as *A. Thalassium*, *A. pelagium*,* A. putridalgicola*,* and A. kapii*, (Ishikawa et al. 2009) a fermentative metabolism could be one suggestion for its tolerance for cyanide inhibition.

Cyanide inhibits the activity of several metalloproteins, including cytochrome c oxidase, due to its strong affinity for iron and other metals (Wibberg et al. 2016). However, cyanotrophs have different strategies to face cyanide toxicity (Cipollone et al. 2007; Roldán et al. 2021). The present isolates possess cyanide poisoning-insensitive alternative oxidases (CIO), the quinol oxidase encoded by the *cioAB* genes, which belong to the cytochrome *bd* family (Arai et al. 2014; Luque-almagro et al. 2005), or lack terminal oxygenases to prevent cyanide poisoning (Beam et al. 2020). in PDN-R2 (*Halomonas stevensii*), cytochrome o ubiquinol oxidase (cytochrome bo oxidase), encoded by the c*ioAB* genes (Arai et al. 2014), was detected.

Effective cyanide-polluted wastewater treatment relies on the synergistic action of cyanide degraders, ammonia oxidizers, and denitrifiers. The present isolates demonstrated this synergy, with most possessing genes associated with both cyanide degradation and denitrification, or with at least one of the two. Particularly, full denitrification genes (*narG*,* nirS*, *norB* and *nosZ)* were detected in three isolates from *Halomonas* (P-DN-B2, P-DN-D2, and P-DN-I2). This suggests that they were denitrifying members of the consortia and supports the notion that denitrification is a significant marker within the genus *Halomonas* (Qu et al. 2011; Quesada et al. 2004). In contrast, our isolate P-DN-R2 (*Halomonas stevensii*) encoded the periplasmic nitrate reductase *napA* and the nitrite reductase *nirB*, which would facilitate assimilation of nitrate as nitrogen source. Interestingly, P-DN-R2 could only grow on solid media when NaNO_3_ was the only nitrogen source and could not grow when it was replaced by NaNO_2_.

The presence of the *narG* and *nrfA* gene in P-CN-AN6 **(***Salisediminibacterium*), indicates it may be able to reduce nitrate to ammonium (Saghaï and Hallin 2024). The *nrfA gene* encodes a subunit of the nitrite ammonification reductase, which catalyzes the direct conversion of nitrite to ammonia. Interestingly, P-DN-P2 (*Alkalibacterium*) which lacked known nitrite reductases and nitrogenases, grew when NaNO_3_/NaNO_2_ was the sole nitrogen source.

Ammonia is one of the intermediate products of cyanide biodegradation (Sharma et al. 2019). The observed nitrite/nitrate generation and subsequent nitrogen removal in the bioreactor may be explained by nitrification, which is the process by which ammonia is transformed into nitrite and nitrate and then followed by denitrification. However, the ammonia monooxygenases and canonical nitrifiers that are essential for this process were not detected in the metagenome. This implies that nitrifying bacteria may experience toxic stress due to the high pH in the reactor (Ni et al. 2023). Heterotrophic nitrification is a possible substitute for autotrophic nitrification. We looked for genes in *Alcaligenes faecalis* that were similar to those connected to nitrification. However, gene clusters with homology to the *dnfABC* gene cluster associated with direct ammonia oxidation (Dirammox) to nitrogen gas (Jq et al. 2022) were not observed. The inability to directly distinguish between biotic and abiotic ammonia oxidation pathways under the experimental conditions represents a limitation of this study.

Heavy metal-resistant bacteria are important candidates for the bioremediation of cyanide-polluted wastewater (Olaya-Abril et al. 2024). Several CDBs have been reported to have heavy metal resistance genes, often clustered together on the genome (Lan et al. 2023). In the present whole genome sequenced isolates, we assessed heavy metal resistance genes and they displayed a prevalence of several heavy metal resistance genes. Seven of our isolates possessed the *arsC* gene, *w*hich encodes arsenate reductase, vital for arsenate reduction(Ali et al. 2013; Kabiraj et al. 2022). Similar to the *arsC* gene, all our isolates harbored *arsA* and *arsB* genes, encoding proteins important for the arsenite efflux pump (Ali et al. 2013). The *arsM* gene, which can encode an enzyme that can methylate arsenite (Biełło et al. 2023) was detected in *Actinotalea*,* Halomonas sp003182195*,* and Halomonas sp024137965*. The presence of both *arsAB* (efflux pump) and *arsM* (methylation) suggested a arsenite detoxification system was present in these isolates.

Copper is an essential transition metal, but its hyperaccumulation affects cellular functions (de Almeida Rodrigues et al. 2022), and some microorganisms detoxify copper when it is beyond their need. Microorganisms detoxify copper by various mechanisms. One of the mechanisms is with the P-type ATPase CopA system (Olaya-Abril et al. 2024). In the present study, all the sequenced isolates, except *Actinotalea*, the copA/B genes that encode a cation efflux pump to expel copper ions from the cell, were detected, indicating their potential for copper resistance.

Similarly, zntA was detected in all isolates, except in *Actinotalea*,* Aliidiomarina*,* and Halomonas sp024137965*, suggesting potential zinc (Zn) homeostasis by involving extrusion of active ATPases encoded by this gene (Blindauer 2015).

## Conclusion

This study demonstrates that alkaliphilic microbial consortia from Ethiopian Rift Valley soda lakes can effectively function in aerobic–anoxic reactors treating cyanide-containing wastewater under high-pH conditions. By combining metagenomic analyses with whole-genome sequencing of isolates, we revealed a metabolically diverse and functionally resilient community capable of degrading cyanide, transforming nitrogen compounds, and tolerating heavy metals. Dominant members, such as those related to *Halomonas*, carried multiple functional traits supporting cyanide degradation, denitrification, and stress resistance, while less abundant isolates, including *Actinotalea* and *Salisediminibacterium*, were captured through cultivation, highlighting the value of complementary approaches. The community displayed multiple enzymatic pathways, cyanide-insensitive respiratory mechanisms, and heavy metal resistance genes, reflecting its adaptability to complex, co-contaminated environments. These findings provide mechanistic insight into how naturally adapted microbial consortia sustain robust wastewater treatment and support their potential application for the bioremediation of cyanide- and metal-laden industrial effluents.

## Data Availability

DNA sequences for the MAGs and isolates are available at Zenodo ([https://doi.org/10.5281/zenodo.16994763](https:/doi.org/10.5281/zenodo.16994763 ))
